# Comparison of dried blood spot and plasma sampling for untargeted metabolomics

**DOI:** 10.1007/s11306-021-01813-3

**Published:** 2021-06-23

**Authors:** Nicole H. Tobin, Aisling Murphy, Fan Li, Sean S. Brummel, Taha E. Taha, Friday Saidi, Maxie Owor, Avy Violari, Dhayendre Moodley, Benjamin Chi, Kelli D. Goodman, Brian Koos, Grace M. Aldrovandi

**Affiliations:** 1 Division of Infectious Diseases, Department of Pediatrics, David Geffen School of Medicine at the University of California, Los Angeles, California; 2 Department of Obstetrics and Gynecology, David Geffen School of Medicine at the University of California, Los Angeles, California; 3 Center for Biostatistics in AIDS Research, Harvard T.H. Chan School of Public Health, Boston, Massachusetts; 4 Department of Epidemiology, Johns Hopkins Bloomberg School of Public Health; 5 UNC Project-Malawi, Kamuzu Central Hospital, Lilongwe, Malawi; 6 MU-JHU Research Collaboration (MUJHU CARE LTD) CRS, Kampala, Uganda; 7 Perinatal HIV Research Unit, Chris Hani Baragwanath Hospital, Soweto, South Africa; 8 Centre for AIDS Research in South Africa; 9 Department of Obstetrics and Gynecology, School of Clinical Medicine, University of KwaZulu Natal, Durban, South Africa; 10 Department of Obstetrics and Gynecology, University of North Carolina at Chapel Hill, North Carolina; 11 Metabolon Inc., Morrisville, North Carolina

**Keywords:** Metabolomics, plasma, dried blood spots, comparison

## Abstract

**Introduction.:**

Untargeted metabolomics holds significant promise for biomarker detection and development. In resource-limited settings, a dried blood spot (DBS)-based platform would offer significant advantages over plasma-based approaches that require a cold supply chain.

**Objectives.:**

The primary goal of this study was to compare the ability of DBS- and plasma-based assays to characterize maternal metabolites. Utility of the two assays was also assessed in the context of a case-control predictive model in pregnant women living with HIV.

**Methods.:**

Untargeted metabolomics was performed on archived paired maternal plasma and dried blood spots from n=79 women enrolled in a large clinical trial.

**Results.:**

A total of 984 named biochemicals were detected across both plasma and DBS samples, of which 627 (63.7%), 260 (26.4%), and 97 (9.9%) were detected in both plasma and DBS, plasma alone, and DBS alone, respectively. Variation attributable to study individual (R^2^=0.54, p<0.001) exceeded that of the sample type (R^2^=0.21, p<0.001), suggesting that both plasma and DBS were capable of differentiating individual metabolomic profiles. Log-transformed metabolite abundances were strongly correlated (mean Spearman rho=0.51) but showed low agreement (mean intraclass correlation of 0.15). However, following standardization, DBS and plasma metabolite profiles were strongly concordant (mean intraclass correlation of 0.52). Random forests classification models for cases versus controls identified distinct feature sets with comparable performance in plasma and DBS (86.5% versus 91.2% mean accuracy, respectively).

**Conclusion.:**

Maternal plasma and DBS samples yield distinct metabolite profiles highly predictive of the individual subject. In our case study, classification models showed similar performance albeit with distinct feature sets. Appropriate normalization and standardization methods are critical to leverage data from both sample types. Ultimately, the choice of sample type will likely depend on the compounds of interest as well as logistical demands.

## Introduction

Untargeted metabolomics has emerged as a powerful technique to investigate the role of metabolites in human health and disease. The unbiased nature of this approach and the ability to characterize novel compounds makes it particularly suitable for tasks such as biomarker discovery and assay development(Carter *et al.*, 2019; Lee and Banerjee, 2020; Sovio *et al.*, 2020). Metabolomic protocols have been developed for diverse sample matrices including serum, plasma, urine, and tissue(Chamberlain *et al.*, 2019; Ly *et al.*, 2016). A major caveat to these approaches is the requirement for significant laboratory infrastructure or a cold supply chain to minimize variation in metabolic profiling due to differences in pre-processing conditions(Chamberlain *et al.*, 2019; Gertsman and Barshop, 2018; La Frano *et al.*, 2018; Wang *et al.*, 2018a; Wang *et al.*, 2018b).

Dried blood spot (DBS) sampling is routinely used for genetic screening and offers the additional advantages of relatively simple collection and ambient storage(Dorsey and Puck, 2019; Greenman *et al.*, 2015; Jansen *et al.*, 2016). DBS-based untargeted metabolomics assays have been previously reported(Koulman *et al.*, 2014; Li *et al.*, 2020; Palmer *et al.*, 2019; Petrick *et al.*, 2017) and used to identify markers of hereditary anemias(van Dooijeweert *et al.*, 2019), newborn birth weight and ethnicity(Petrick *et al.*, 2017), and inborn errors of metabolism(Haijes *et al.*, 2019).

Here, we used paired maternal plasma and DBS samples to assess the viability of DBS as a matrix for untargeted metabolomics. We characterized the specific compounds uniquely identified by each assay as well as those that were differentially abundant between the two assays. We also used intraclass correlation (ICC) to assess agreement between the two assays and identified a set of compounds with high reproducibility as potential candidates for DBS-based biomarker assays. As a proof of principle, we characterized plasma and DBS-derived signatures in a case-control cohort of women living with HIV.

## Methods

### Study population and sample collection

Plasma and dried blood spot (DBS) samples were obtained from n=79 pregnant women living with HIV as part of a larger clinical trial(Fowler *et al.*, 2016). Samples were collected during pregnancy either prior to antiretroviral initiation (untreated) or during treatment with either a single drug (zidovudine) or protease-inhibitor based antiretroviral therapy (PI-ART). For the purposes of this study, women were labeled as case or control by the larger trial so the case study could be performed in a statistically blinded manner.

Blood samples were collected in BD Vacutainer ACD tubes and transferred to Whatman 903 Protein Saver Cards. Cards were then air dried for at least four hours and then placed into gas impermeable bags with a desiccant pack and humidity card at −20°C or colder. DBS cards were transferred to a biorepository for long term storage at −80°C until assayed. The remaining blood volume was centrifuged at 400 × g for 10 minutes. Plasma was transferred to a new sterile tube and centrifuged again at 800 × g for 10 minutes. Aliquots were taken and placed into sterile cryovials for storage at −80°C.

### Sample processing

Samples were processed by Metabolon Inc. according to published methods with modifications as described for DBS samples(Evans *et al.*, 2014; Evans *et al.*, 2009; Ford *et al.*, 2020). Briefly, samples were prepared using the automated MicroLab STAR^®^ system from Hamilton Company. Several recovery standards were added prior to the first step in the extraction process for QC purposes. For DBS samples, 2 × 6mm punches were extracted per sample; one punch taken from the middle of two spots, then combined in the extraction plate. These were then shaken vigorously with a small aliquot of water to reconstitute the dried sample. Subsequently, DBS and plasma samples were extracted using the same procedure. To remove protein, dissociate small molecules bound to protein or trapped in the precipitated protein matrix, and to recover chemically diverse metabolites, proteins were precipitated with methanol under vigorous shaking for 2 min (Glen Mills GenoGrinder 2000) followed by centrifugation. The resulting extract was divided into five fractions: two for analysis by two separate reverse phase (RP)/UPLC-MS/MS methods with positive ion mode electrospray ionization (ESI), one for analysis by RP/UPLC-MS/MS with negative ion mode ESI, one for analysis by HILIC/UPLC-MS/MS with negative ion mode ESI, and one sample was reserved for backup. Samples were placed briefly on a TurboVap^®^ (Zymark) to remove the organic solvent. The sample extracts were stored overnight under nitrogen before preparation for analysis.

### Ultrahigh performance liquid chromatography-tandem mass spectroscopy (UPLC-MS/MS)

Untargeted ultra-high-performance liquid chromatography/tandem mass spectrometry of known biochemicals was conducted on plasma and DBS samples by Metabolon Inc. according to published methods(Evans *et al.*, 2014; Evans *et al.*, 2009; Ford *et al.*, 2020). All methods utilized a Waters ACQUITY ultra-performance liquid chromatography (UPLC) and a Thermo Scientific Q-Exactive high resolution/accurate mass spectrometer interfaced with a heated electrospray ionization (HESI-II) source and Orbitrap mass analyzer operated at 35,000 mass resolution. The sample extract was dried then reconstituted in solvents compatible to each of the four methods. Each reconstitution solvent contained a series of standards at fixed concentrations to ensure injection and chromatographic consistency. One aliquot was analyzed using acidic positive ion conditions, chromatographically optimized for more hydrophilic compounds. In this method, the extract was gradient eluted from a C18 column (Waters UPLC BEH C18–2.1×100 mm, 1.7 μm) using water and methanol, containing 0.05% perfluoropentanoic acid (PFPA) and 0.1% formic acid (FA). Another aliquot was also analyzed using acidic positive ion conditions, however it was chromatographically optimized for more hydrophobic compounds. In this method, the extract was gradient eluted from the same aforementioned C18 column using methanol, acetonitrile, water, 0.05% PFPA and 0.01% FA and was operated at an overall higher organic content. Another aliquot was analyzed using basic negative ion optimized conditions using a separate dedicated C18 column. The basic extracts were gradient eluted from the column using methanol and water, however with 6.5mM Ammonium Bicarbonate at pH 8. The fourth aliquot was analyzed via negative ionization following elution from a HILIC column (Waters UPLC BEH Amide 2.1×150 mm, 1.7 μm) using a gradient consisting of water and acetonitrile with 10mM Ammonium Formate, pH 10.8. The MS analysis alternated between MS and data-dependent MSn scans using dynamic exclusion. The scan range varied slighted between methods but covered 70–1000 m/z.

### Data analysis

Compounds were identified by Metabolon Inc. as previously described(Evans *et al.*, 2014; Evans *et al.*, 2009; Ford *et al.*, 2020). Briefly, compounds were identified by comparison of experimental data to a library of authentic standards using accurate mass, retention time and fragmentation spectrum(Sumner *et al.*, 2007). Technical replicates of a DBS QC sample, that had been prepared in bulk at Metabolon using a single lot of whole blood to spot a large number of cards contemporaneously, were extracted in each 48-sample plate and injected periodically throughout the platform run to monitor the overall process variability of endogenous biochemicals. The internal standards that were added to each sample immediately before analysis (spiked in the reconstitution solvents) were monitored across all experimental samples to assess instrument variability. Overall process variability met Metabolon’s acceptance criteria in all plasma and DBS sample sets (< 10% and < 15% median RSD, respectively). Recovery standards (RS) were added to every sample (spiked in the crash solvent) at the beginning of the extraction and used to monitor variability in the extraction process. They were specifically chosen because they are highly reproducible and are detected well on each of the four analytical methods. RS plots of mean-scaled response per sample were checked to ensure all responses were within 15% of the mean.

Raw area under the curve (AUC) values from plasma and DBS samples were used to tabulate the number of compounds quantified in each sample type. For all other analyses, missing values were imputed with the minimum quantified value of each biochemical in the sample matrix (plasma or DBS). All values were log-transformed and standardized using a Z-transform. Log-transformed values refer to those following imputation and log transformation but prior to standardization. Standardized values refer in effect to Z scores.

Analyses were conducted in the R statistical environment version 3.6.1(R Core Team, 2019). Permutational multivariate analysis of variance (PERMANOVA) with Euclidean distances as implemented in the ‘vegan’ package was utilized to assess variance attributable to study individual and sample type. Two-way (absolute agreement) intraclass correlation between paired plasma and DBS measurements was calculated for each compound using the ‘psych’ package(Revelle, 2019). Multiple testing correction using the Benjamini-Hochberg false discovery rate method was utilized as appropriate and an adjusted p-value of <0.05 was considered significant(Benjamini and Hochberg, 1995).

Random forests classification models (‘randomForest’ package)(Liaw and Wiener, 2002) were constructed separately for women prior to treatment (untreated) or during treatment with either zidovudine monotherapy or protease inhibitor-based ART. Three of the women were exposed to other ART regimens and were excluded from this analysis. Plasma or DBS metabolite abundances were used as covariates with a binary outcome for case versus control. One hundred forests each comprising 10,000 trees were used to obtain mean feature importance values and a sparse feature set was subsequently identified by 10-fold cross validation. Sparse models were then constructed with the selected number of features and used to calculate all reported performance metrics.

Analysis code and data files necessary to reproduce the analyses are available at https://github.com/AldrovandiLab/DBSvPlasma-metabolomics.

## Results and discussion

### Study population

The study participants were divided into cases/controls and by the treatment regimen at the time of specimen collection(Fowler *et al.*, 2016). Paired plasma and DBS samples were drawn from pregnant women living with HIV either prior to treatment (untreated) or on one of two treatment arms (zidovudine monotherapy or protease inhibitor-based ART). Baseline characteristics of the n=79 participants are shown in Table 1.

### Compound detection from plasma and DBS samples

**Figure 1:**
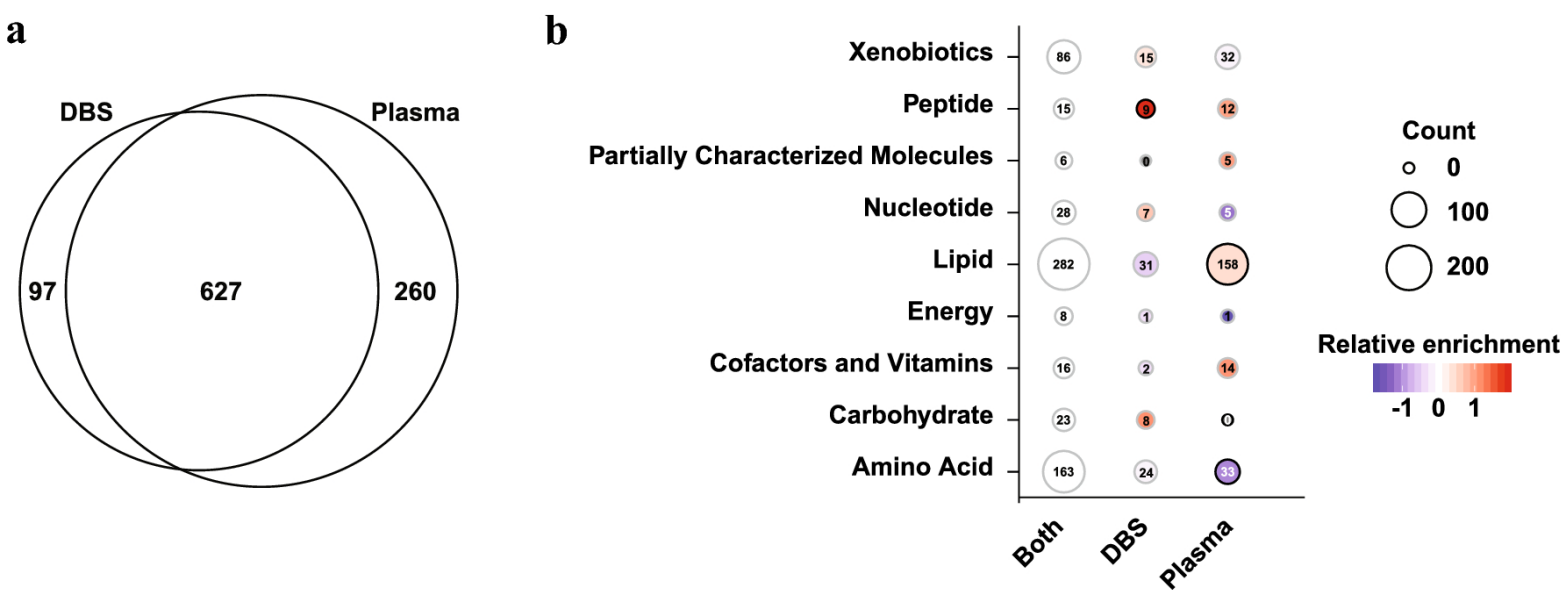
Detection of compounds from plasma and dried blood spots. (a) Venn diagram showing the number of compounds detected by both assays, or a single assay. (b) Breakdown of compounds detected by both assays or either assay alone into specific classes. Circle size is proportional to the absolute number of compounds detected and color shading shows enrichment or depletion of the specific class in the DBS and Plasma alone columns relative to the proportion detected by both assays. Black borders indicate significant differences by Chi-square test (adjusted p < 0.05).

A total of 984 named compounds were detected across both plasma and DBS samples, of which 627 (63.7%), 260 (26.4%), and 97 (9.9%) were detected in both plasma and DBS, plasma alone, and DBS alone, respectively (Figure 1A, Online Resource 1). Most compounds were detected broadly across the samples, with 905 (92.0%) being detected in at least half of the 79 samples and 577 (58.6%) being detected in all of the samples (Online Resource 2). Of the 7 compounds that were detected in fewer than 3 samples, six were drugsfound in both DBS and plasma from the same study individual (Online Resource 1). These are therefore unlikely to represent artifacts from the sample matrix itself. In contrast, androsterone glucuronide was only detected in 2 DBS samples but 77 plasma samples, suggesting that it may not be easily quantified by DBS. Overall, lipids were preferentially detected in plasma but not DBS (Figure 1B, χ^2^ p<0.001 versus compounds detected in both plasma and DBS). Peptides, specifically dipeptides, were more likely to be detected in DBS alone (Figure 1B, χ^2^ test p=0.001 versus compounds detected in both).

### Measurement consistency and effect of standardization

**Figure 2:**
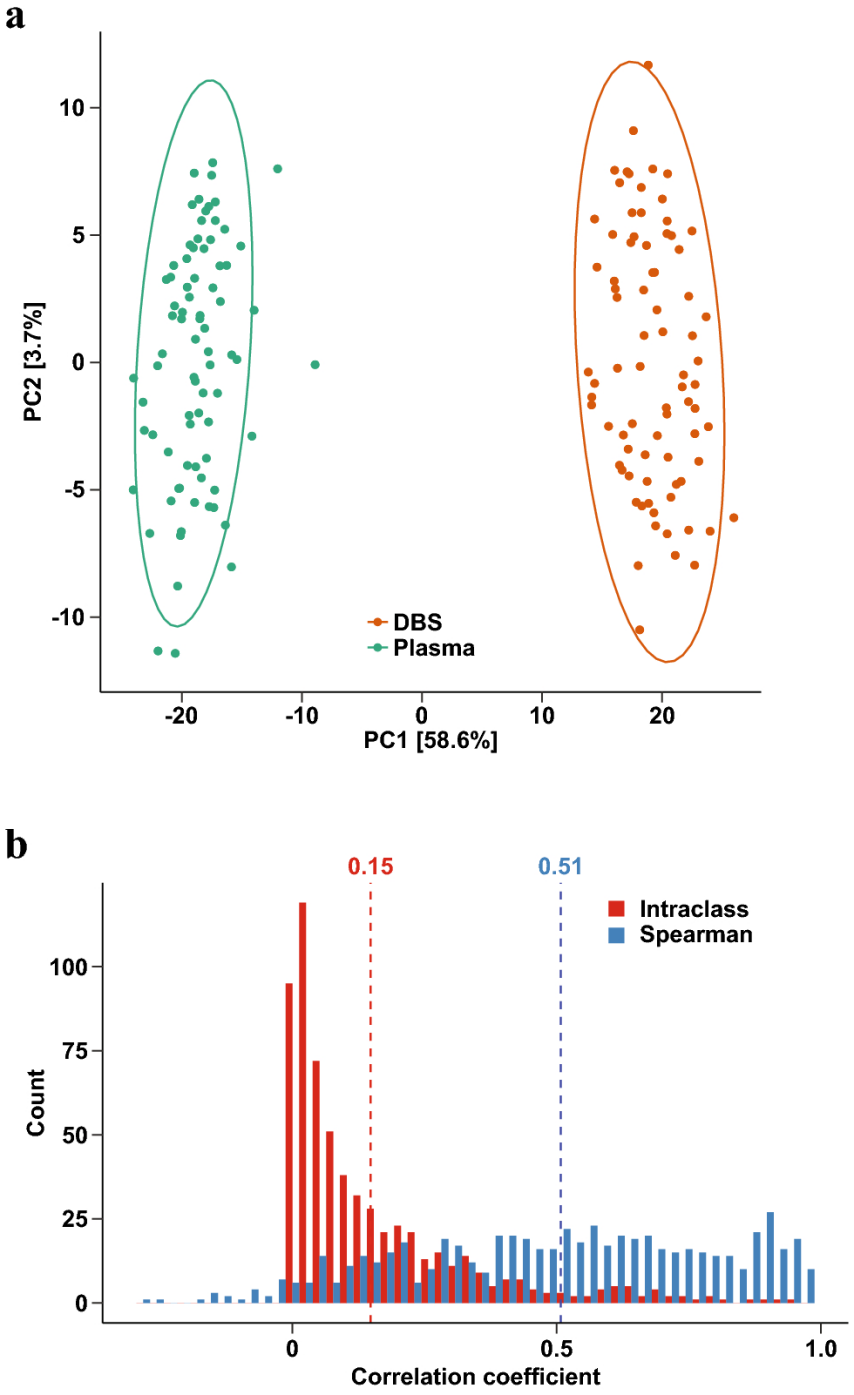
Consistency between log-transformed DBS and plasma metabolite profiles. (a) PCA plot of log-transformed metabolite profiles. Ellipses show 95% confidence regions for each sample type. Numbers in brackets denote the percentage of total variation explained by each principal component. (b) Distribution of interclass correlation (red) and Spearman correlation (blue) coefficients between paired plasma and DBS samples. Dotted lines denote means.

We next wanted to assess the consistency of metabolomic profiles obtained from the paired plasma and DBS samples. Based on principal coordinates analysis with Euclidean distances, log-transformed but not standardized metabolomic profiles were markedly different between paired plasma and DBS samples (Figure 2A). Permutational multivariate analysis of variance (PERMANOVA) identified sample type (R^2^=0.57, p<0.001) and study individual (R^2^=0.22, p<0.001) to be significant drivers of overall variation in metabolomic profiles. Spearman correlation coefficients skewed positive (mean rho=0.51) but intraclass correlation coefficient (ICC) values showed minimal agreement between the paired DBS and plasma samples (mean ICC=0.15, Figure 2B). This is likely due to the ability of ICC to account for bias in the data values. These results suggest that unstandardized DBS metabolite abundances are correlated with but significantly biased from their plasma counterparts.

**Figure 3:**
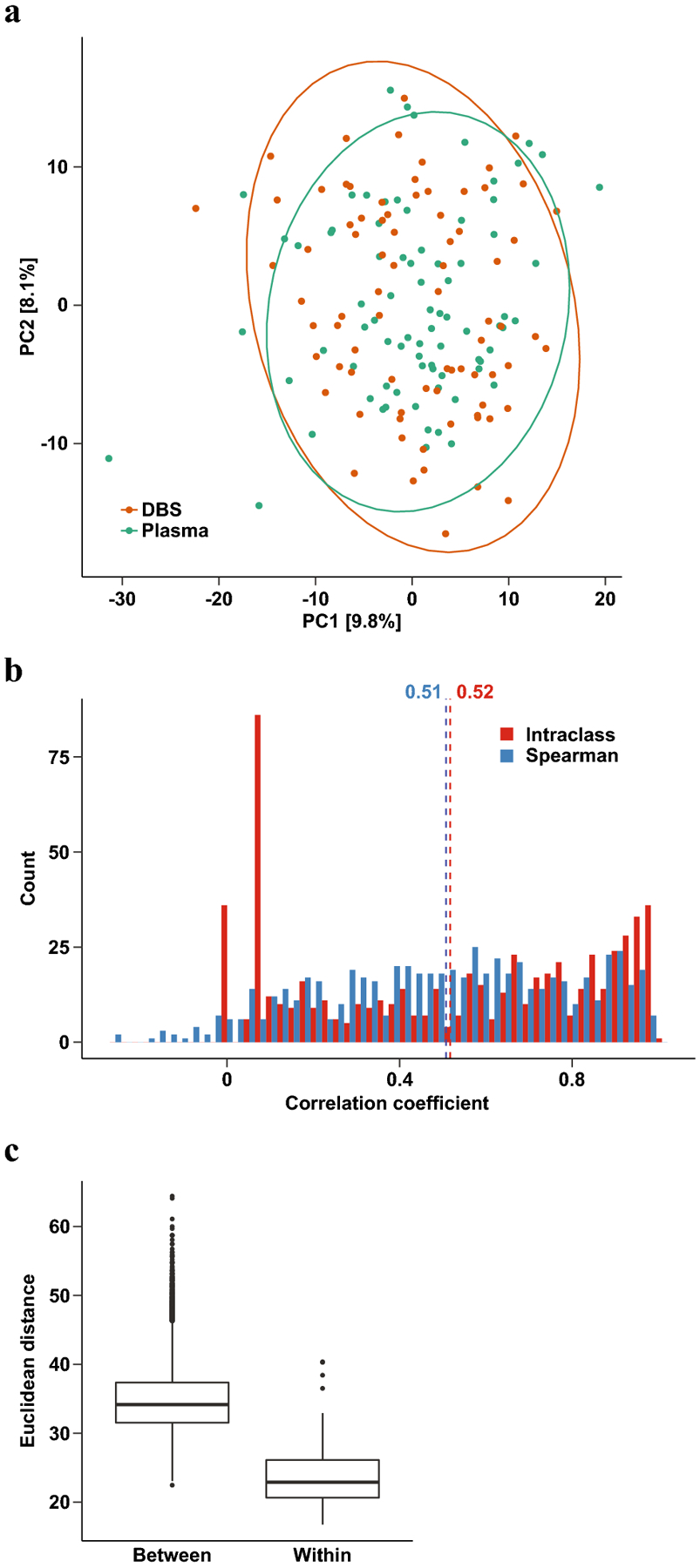
Consistency between log-transformed and standardized DBS and plasma metabolite profiles. (a) PCA plot of log-transformed and standardized metabolite profiles. Ellipses show 95% confidence regions for each sample type. Numbers in brackets denote the percentage of total variation explained by each principal component. (b) Distribution of interclass correlation (red) and Spearman correlation (blue) coefficients between paired plasma and DBS samples. Dotted lines denote means. (c) Boxplot of Euclidean distances between plasma and DBS samples across different women (‘Between’) or within the same woman (‘Within’).

Given the observed bias, we decided to repeat the above consistency analyses using standardized values of the DBS and plasma metabolite abundances. As expected, standardized profiles did not display the same distinct separation by sample type (Figure 3A), and PERMANOVA attributed 69% (p<0.001) of the overall variation to the study individual. Furthermore, Spearman correlation coefficients were unchanged but ICC values were dramatically increased (mean ICC=0.52, Figure 3B). Distances between plasma and DBS samples from the same participant were significantly smaller than those from different participants (Wilcoxon p<0.001, Figure 3C). Altogether, these results show that simple standardization of DBS metabolite profiles removes the observed bias compared to plasma-derived profiles while retaining inter-individual differences. These results support the conclusion that appropriate statistical treatment of metabolomic data derived from both DBS and plasma sample types can allow for their use in joint modeling or classification tasks.

**Figure 4:**
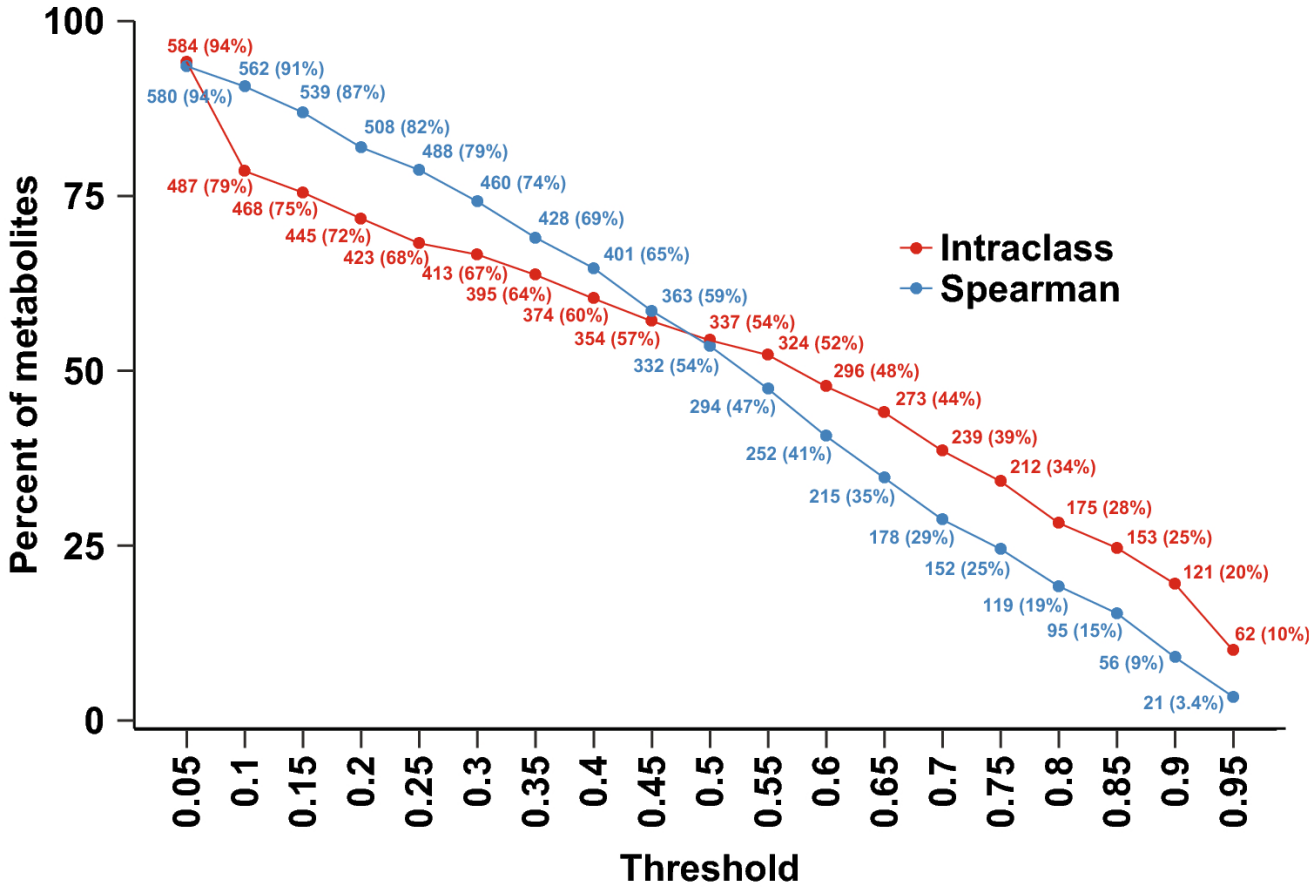
Number of compounds with Spearman (red) or intraclass (blue) correlation coefficients above the specified threshold.

Another important consideration is whether DBS- and plasma-derived metabolomic profiles report consistent measurements for specific compounds of interest. A relatively small subset of compounds was found to demonstrate good (ICC >= 0.75, n=212) or excellent (ICC >= 0.9, n=121) reproducibility (Figure 4 and Online Resource 3). Compounds classified as xenobiotics were increasingly enriched with higher ICCs (χ^2^ p<0.001 and p=0.001 for compounds with ICC >= 0.75 and 0.9 versus all compounds, Online Resource 4), suggesting that these compounds show especially high agreement between plasma- and DBS-based assays. However, these compounds are not likely to be particularly distinctive to pregnant women so their suitability as biomarkers is limited in this context. Indeed, two of these (trimethoprim and N4-acetyl-5-hydroxysulfamethoxazole) are a common antibiotic treatment for urinary tract and skin and soft tissue infections(Bowen *et al.*, 2017; Mehnert-Kay, 2005), and a third (2,6-dihydroxybenzoic acid) is a metabolite of the commonly used topical agent salicylic acid(Wishart *et al.*, 2018). On the other hand, several of the highly reproducible lipids (e.g. androsterone sulfate, dehydroepiandrosterone sulfate (DHEA-S), estrone 3-sulfate, glycocholate) are known pregnancy-associated hormones that have been reported in other studies using dried blood spot sampling(Janzen *et al.*, 2010; Petrick *et al.*, 2017).

### Case study in pregnant women living with HIV

As a proof of concept for the utility of DBS metabolomics, we next attempted to distinguish cases versus controls in pregnant women living with HIV who were either untreated or on either a zidovudine monotherapy or PI-based ART regimen. Using a standard random forests classification approach (see Methods), we constructed sparse models in each of the three treatment groups and sample matrices. DBS-based models achieved accuracies of 90.9%, 95.7%, and 87.1% for the untreated, zidovudine monotherapy, and PI-based ART groups, respectively. Plasma-based models achieved slightly lower accuracies of 86.4%, 91.3%, and 77.4%. However, given the relatively small sample sizes, it is difficult to draw meaningful conclusions from these performance metrics.

**Figure 5:**
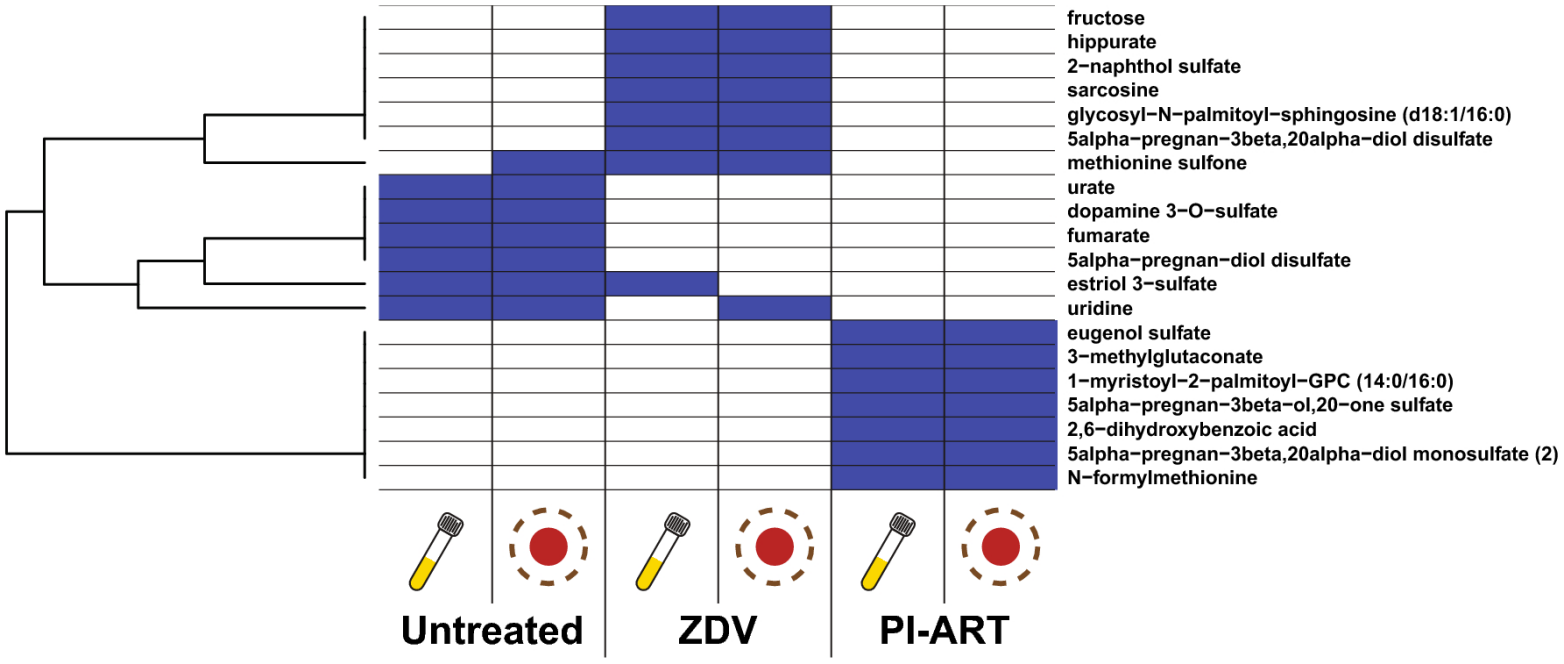
Features that are consistently selected in random forests models from both plasma and DBS metabolite profiles. The specific drug regimen is noted at the bottom. Each column represents an independent model within the indicated sample matrix and drug regimen. Only features selected in at least 2 models are shown, and shaded cells denote selected features.

Overall, the feature sets identified using the sparse modeling approach were strikingly different between plasma and DBS (Online Resources 5 and 6). In fact, only 20/147 total features selected in any of the models were selected in both plasma- and DBS-based models for the same treatment regimen (Figure 5 and Online Resource 6). This subset of compounds showed significantly higher concordance (Wilcoxon p<0.001, Online Resource 7) and includes a number of sulfated steroid compounds as well as several metabolites involved in methionine metabolism (methionine sulfone and N-methylmethionine). Given the relatively small sample size, however, it is worth emphasizing that this case study is not intended as a conclusive analysis on pregnant women living with HIV but rather a preliminary study on the use of plasma- and DBS-based metabolomics for predictive modeling and biomarker discovery.

## Conclusion

In this study, we compared the utility of plasma and dried blood spot (DBS) sampling for untargeted metabolomics profiling of pregnant women living with HIV. Following appropriate standardization, the two sample matrices had distinct features or combinations of features that are potential case-associated biomarkers. Selection of sampling strategy for any particular study should be based on the likelihood of capturing the biomarkers of interest in the sample type as well as resource availability.

## Supplementary Material

1723975_Ol_tab1 **Online Resource 1** Compounds detected by plasma and DBS assays.

1723975_Ol_Fig2 **Online Resource 2** Number of compounds by plasma and DBS assays as a function of detection threshold. Numbers on x-axis indicate the number of samples in which a compoundsneeds to be detected in order to be counted.

1723975_Ol_Fig3 **Online Resource 3** Compounds with highly reproducible profiles between the two assays (ICC >= 0.9). ‘ICC’, ‘p’, ‘padj’, ‘lower’, and ‘upper’ columns refer to estimates and p-values associated with intraclass correlation. ‘rho’, ‘spearman_p’, and ‘spearman_padj’ columns refer to Spearman correlation coefficients and p-values.

1723975_Ol_Fig4 **Online Resource 4** Superpathway distribution of highly reproducible compounds meeting the specified ICC threshold.

1723975_Ol_Fig5 **Online Resource 5** Features selected by RF models. Columns with TRUE/FALSE values denote whether each feature was selected in the corresponding RF model. ‘ICC’, ‘p’, ‘padj’, ‘lower’, and ‘upper’ columns refer to estimates and p-values associated with intraclass correlation. ‘rho’, ‘spearman_p’, and ‘spearman_padj’ columns refer to Spearman correlation coefficients and p-values.

1723975_Ol_Fig6 **Online Resource 6** Heatmap of all features among the random forests models. Shaded cells show the mean feature importance for the indicated model as described in the methods.

1723975_Ol_Fig7 **Online Resource 7** Boxplot of ICC values in consistently selected RF features (selected in both plasma and DBS models for any regimen) versus all other RF features.

## Figures and Tables

**Table 1:** Demographics of study participants.

	Case untreated	Case ZDV	Case PI-ART	Case other	Control untreated	Control ZDV	Control PI-ART	Control other	p
**n**	11	13	16	1	11	10	15	2	
**Country (%)**									0.161
India	0 (0.0)	0 (0.0)	2 (12.5)	0 (0.0)	1 (9.1)	0 (0.0)	1 (6.7)	0 (0.0)	
Malawi	3 (27.3)	8 (61.5)	4 (25.0)	0 (0.0)	4 (36.4)	7 (70.0)	0 (0.0)	1 (50.0)	
South Africa	6 (54.5)	4 (30.8)	8 (50.0)	1 (100.0)	6 (54.5)	2 (20.0)	10 (66.7)	1 (50.0)	
Uganda	2 (18.2)	1 (7.7)	1 (6.2)	0 (0.0)	0 (0.0)	1 (10.0)	3 (20.0)	0 (0.0)	
Zambia	0 (0.0)	0 (0.0)	1 (6.2)	0 (0.0)	0 (0.0)	0 (0.0)	1 (6.7)	0 (0.0)	
									
**Gestational age at sample collection, weeks (mean (SD))**	29.62 (2.78)	30.19 (2.95)	31.51 (2.21)	32.00 (NA)	31.80 (1.84)	30.40 (2.53)	30.56 (1.86)	28.07 (7.18)	0.213
**Gestational age at delivery, weeks (mean (SD))**	32.13 (3.15)	32.96 (2.71)	33.76 (1.58)	35.43 (NA)	41.16 (4.36)	39.94 (2.66)	39.90 (2.13)	39.78 (2.93)	<0.001
**Infant sex, male (n (%))**	4 (36.4)	4 (30.8)	8 (50.0)	1 (100.0)	3 (27.3)	6 (60.0)	10 (66.7)	1 (50.0)	0.248

## Data Availability

The data cannot be made publicly available due the ethical restrictions in the study’s informed consent documents and in the International Maternal Pediatric Adolescent AIDS Clinical Trials (IMPAACT) Network’s approved human subjects protection plan; public availability may compromise participant confidentiality. However, data are available to all interested researchers upon request to the IMPAACT Statistical and Data Management Center’s data access committee (sdac.data@fstrf.org) with the agreement of the IMPAACT Network.
