# Supplementary figures and images for: Comparison of dried blood spot and plasma sampling for untargeted metabolomics
Source: Metabolomics. Author manuscript; Available in PMC 2022 Jun 23. (PMC8340475; doi:10.1007/s11306-021-01813-3)

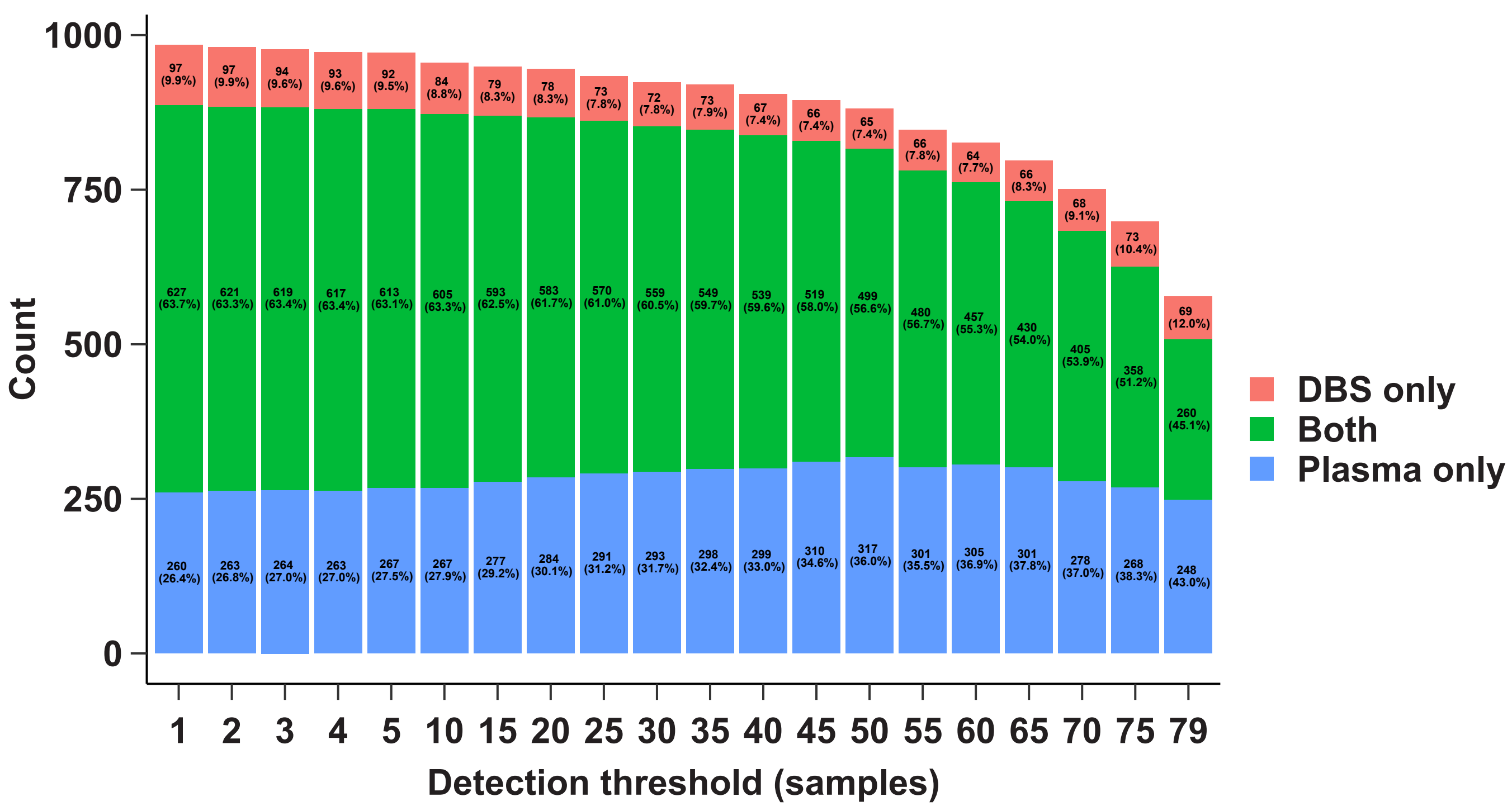

Supplement: 1723975_Ol_Fig2 — Online Resource 2 Number of compounds by plasma and DBS assays as a function of detection threshold. Numbers on x-axis indicate the number of samples in which a compoundsneeds to be detected in order to be counted. [file NIHMS1723975-supplement-1723975_Ol_Fig2.pdf]

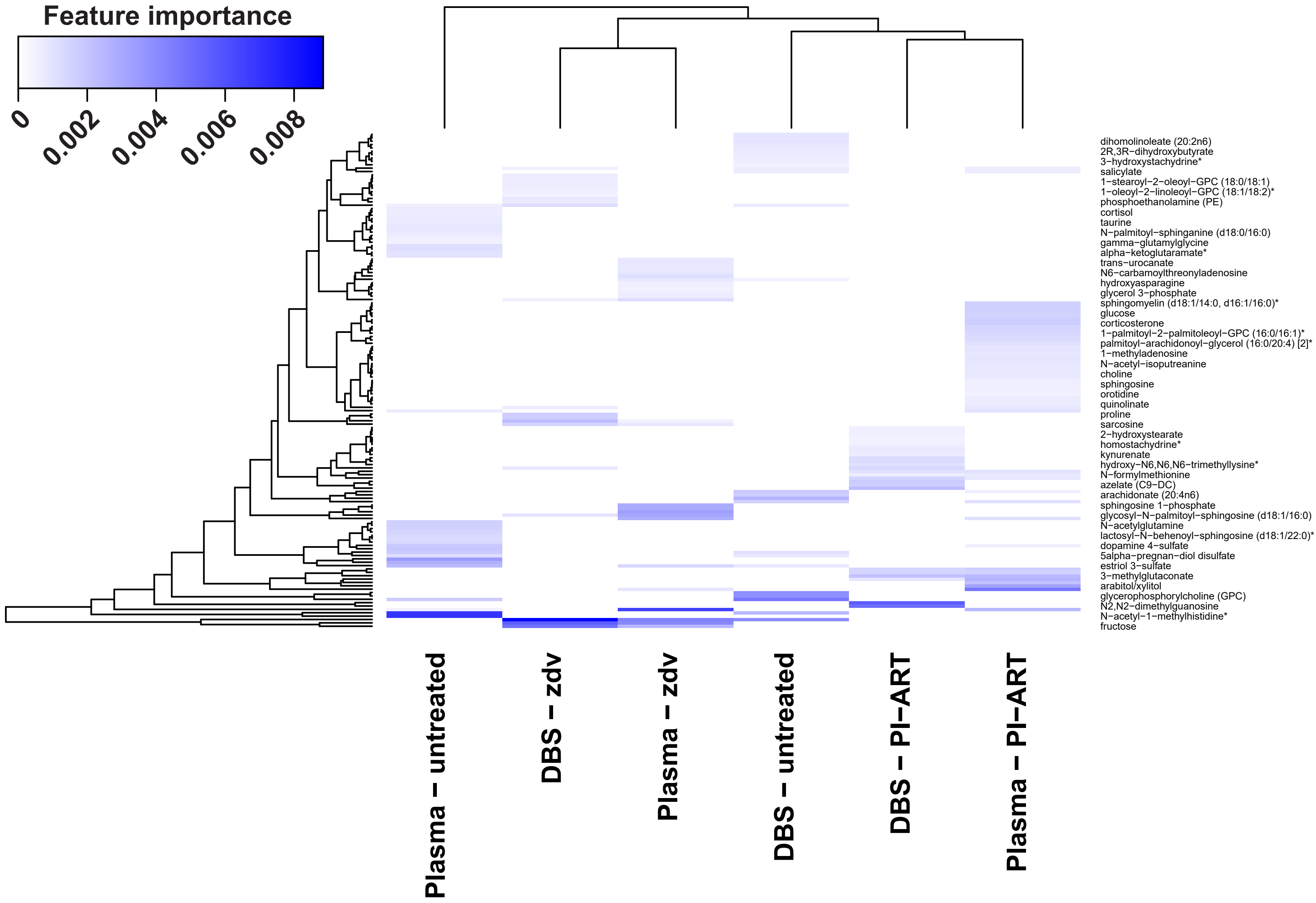

Supplement: 1723975_Ol_Fig6 — Online Resource 6 Heatmap of all features among the random forests models. Shaded cells show the mean feature importance for the indicated model as described in the methods. [file NIHMS1723975-supplement-1723975_Ol_Fig6.pdf]

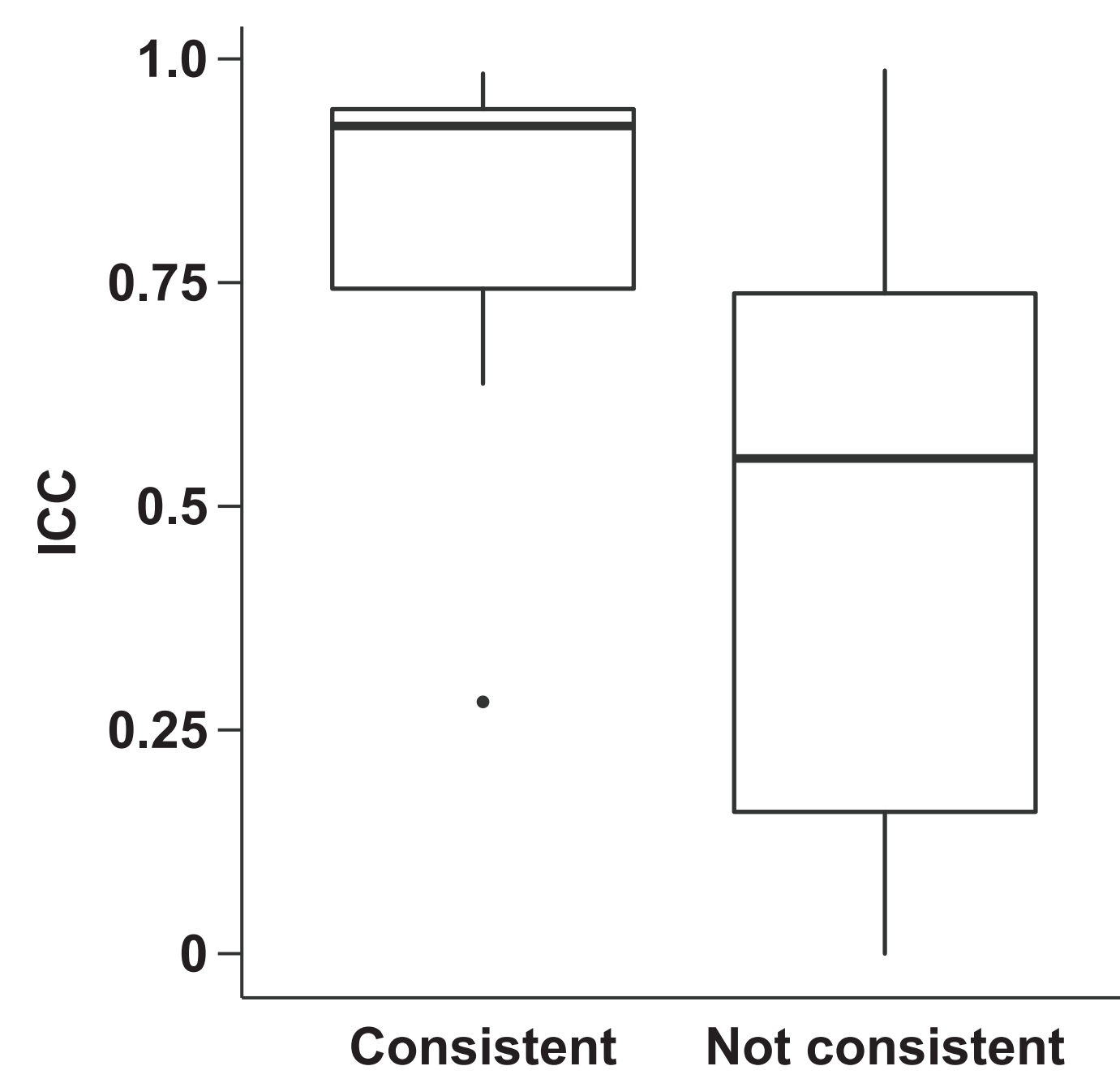

Supplement: 1723975_Ol_Fig7 — Online Resource 7 Boxplot of ICC values in consistently selected RF features (selected in both plasma and DBS models for any regimen) versus all other RF features. [file NIHMS1723975-supplement-1723975_Ol_Fig7.pdf]
