# Supplementary material for: Comparison of dried blood spot and plasma sampling for untargeted metabolomics
Source: Metabolomics. Author manuscript; Available in PMC 2022 Jun 23. (PMC8340475; doi:10.1007/s11306-021-01813-3)
Supplement: 1723975_Ol_Fig4 — Online Resource 4 Superpathway distribution of highly reproducible compounds meeting the specified ICC threshold. [file NIHMS1723975-supplement-1723975_Ol_Fig4.pdf]

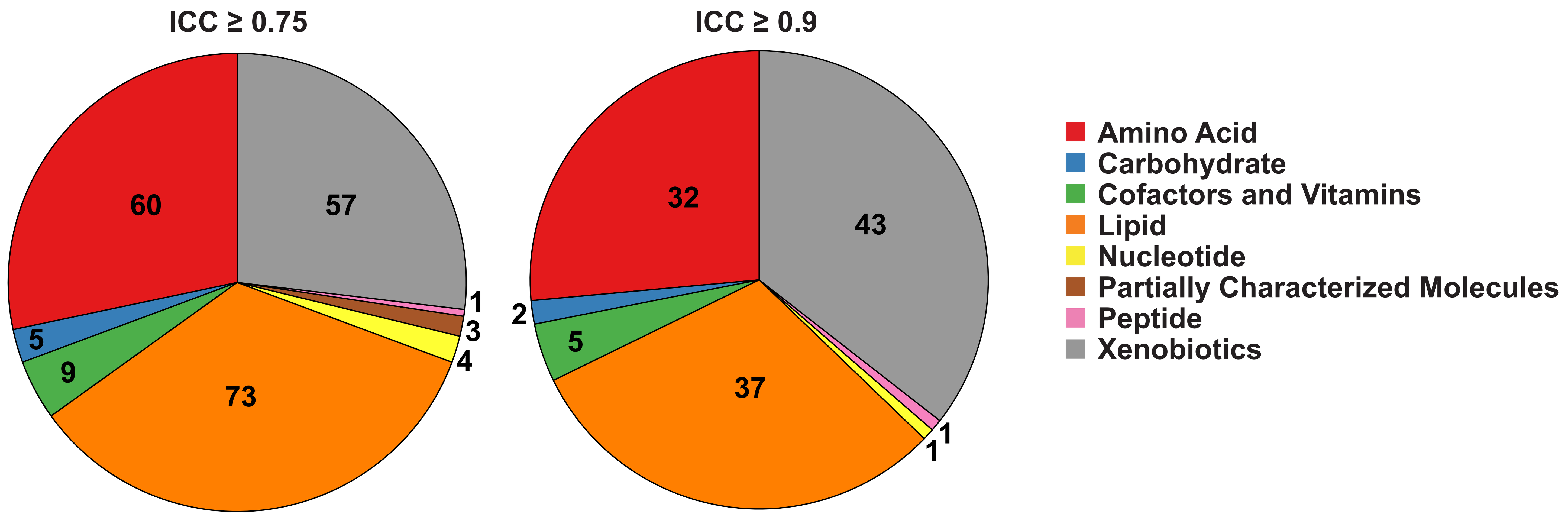
ICC ≥ 0.75
60
57
1
3
4
5
9
73
ICC ≥ 0.9
32
43
2
5
37
1
1
Amino Acid
Carbohydrate
Cofactors and Vitamins
Lipid
Nucleotide
Partially Characterized Molecules
Peptide
Xenobiotics
